# Linear and Volumetric Analysis of Maxillary Sinus Pneumatization in a Sri Lankan Population Using Cone Beam Computer Tomography

**DOI:** 10.1155/2021/6659085

**Published:** 2021-04-08

**Authors:** Pilana Vithanage Kalani Shihanika Hettiarachchi, Pulihinga Mudiyanselage Prabhath Chaminda Gunathilake, Rasika Manori Jayasinghe, Manil Christopher Fonseka, Ranasinghe Mudiyanselage Wikum Roshan Bandara, Chinthani Deepthi Nanayakkara, Ruwan Duminda Jayasinghe

**Affiliations:** ^1^Department of Oral Medicine and Periodontology, Faculty of Dental Sciences, University of Peradeniya, Sri Lanka; ^2^Department of Statistics and Computer Science Faculty of Science, University of Peradeniya, Sri Lanka; ^3^Department of Prosthodontics, Faculty of Dental Sciences, University of Peradeniya, Peradeniya, Sri Lanka; ^4^Department of Restorative Dentistry, Faculty of Dental Sciences, University of Peradeniya, Peradeniya, Sri Lanka; ^5^Department of Basic Sciences, Faculty of Dental Sciences, University of Peradeniya, Sri Lanka

## Abstract

**Objective:**

The objectives of this study were to evaluate the linear and volumetric measurements of the maxillary sinus in relation to sex and side on cone beam computer tomographic (CBCT) images in a Sri Lankan population.

**Methods:**

A total of 20 sets of CBCT images selected from the database at the Division of Oral Medicine and Radiology, Faculty of Dental Sciences, University of Peradeniya, Sri Lanka, were evaluated. Linear measurements were obtained in a craniocaudal (height), anteroposterior (length), and mediolateral (width) dimensions. Volume was computed by using the same data using a computerized 3D modeling software developed for 3D measurements and calculations.

**Results:**

The maximum mean craniocaudal dimension was at the level of the 1st and the 2nd molar tooth bilaterally. The largest average craniocaudal, mediolateral, and anteroposterior extensions of the maxillary sinus using CBCT were 31.71 ± 5.44 mm, 21.28 ± 5.09 mm, and 32.92 ± 4.31 mm, respectively. The differences between the sides and sex showed no statistical significance (*P* > 0.05), except for the maximum average value in craniocaudal dimension which showed a statistically significant difference in relation to gender (*P* = 0.02).

**Conclusion:**

There is no significant difference in the largest average craniocaudal, mediolateral, and anteroposterior extensions of the maxillary sinus when gender and side were compared. However, the maximum average value in craniocaudal dimension had a statistically significant difference in relation to gender. This study provides valuable knowledge of the anatomical dimensions of the maxillary sinus which may help clinicians in treatment planning.

## 1. Introduction

Dental implant placement has revolutionized the dentistry in the recent past has resulted in transforming our skills as oral health care practitioners. The censorious structure within the posterior mandible is the inferior alveolar nerve which must be avoided when an implant is placed. Similarly, the sinus is the important structure situated within the maxilla, which can limit the utilization of implants causing complications.

The largest of the paranasal sinuses is the maxillary sinuses, and it was first described by Leonardo da Vinci in 1489 and later documented as “antrum of Highmore” following its description by an English anatomist, Nathaniel Highmore in 1651 [1, 2].

The volume of this pyramidal shape structure usually measures 15 cc; however, the value may vary with age, gender, and ethnicity [3, 4]. In addition, dimensions of the maxillary sinus tend to change due to loss or absence of teeth as well. Identification of proper linear and volumetric measurements of the maxillary sinus in a given population is clinically important as it can guide the clinician in proper decision-making during various treatment procedures. Cone-beam computed tomography (CBCT), since the introduction into routine clinical practice, is frequently used to evaluate sinus anatomy especially prior to dental implant treatment [5, 6]. It facilitates evaluation of the maxillofacial region with high-geometric-accuracy data, isotropic voxel values, and short scanning times [5, 7]. In addition, it provides increased precision, lower radiation doses, and lower costs than computed tomography (CT) [7, 8]. Further, CBCT is an efficient alternative to standard multidetector computed tomography (MDCT) to identify sinusitis as the image quality and radiation dose are comparable to MDCT [9].

In spite of being an important anatomical structure, limited availability of literature together with marked population variations [10] warrants the necessity to evaluate parameters in relation to maxillary sinus especially in the local population. Hence, the present study was undertaken to ascertain the linear and volumetric measurements of the maxillary sinus in relation to sex and side on CBCTs in a Sri Lankan population.

## 2. Methods

This study was performed as a retrospective analysis of data obtained from the archives of the Division of Oral Medicine and Radiology of the Dental Teaching Hospital, Peradeniya, Sri Lanka. Deidentified images of twenty maxillary sinuses belonging to 10 patients between the 20-30 years of age were analyzed to determine the dimensions of the maxillary sinuses. Images of patients with incomplete clinical records, distorted images, previous history of maxillary surgery, gross malocclusions, or cranio-facial anomalies and patients with cleft lip and palate, maxillofacial trauma, and maxillary arches with edentulous areas involving more than two posterior teeth and sinuses with mucosal thickening were excluded from the study. Ethical clearance was obtained from the Ethics Review Committee, Faculty of Dental Sciences, University of Peradeniya, and written consent had been obtained from all the participants for using the data for study purposes prior to image acquisition.

All CBCT images were acquired using a Vatech, PaX-Duo3D CBCT scanner (Vatech Corporation, South Korea) using a range of 18-200 uSV, 60 to 90 kvp, and 2-15 mA allowing any adjustment within each FOV and voxel size under standard settings. Images were stored and converted to a DICOM format using the acquisition software integrated to the above CBCT machine. The linear measurements were obtained using EzDent software measurement tools with precision values of 0.1 mm, while the area and the volume were computed by using the same DICOM images in a computerized 3D modeling software [11].

### 2.1. Linear Measurement Determination

Parameters described by Hamdy et al. (2014) were mostly used in the study considering the anatomical fact that the maxillary sinus is pyramidal in shape, with an almost square base that is oriented medially [12]. The measurements of the sinus dimensions were conducted using the methods described by Hamdy et al. in 2014 [12] as the guide.

**Figure 1:**
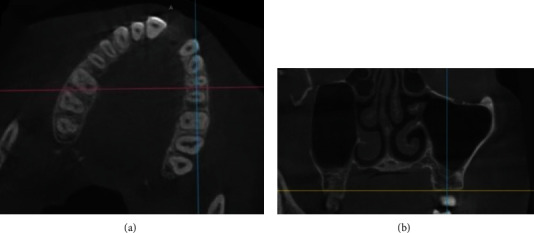
(a) Adjustment of axial cut at area of intended measurement by rotation of the axial image till the orientation axis for the coronal cut (pink line) becomes perpendicular on buccal cortex for each side. (b) Coronal cut revealing the actual craniocaudal measurement conducted along the orientation axis for the sagittal cut (blue line).

1. Craniocaudal (CC) measurements are depicted in Figure 1

Bilateral measurements between upper first and second premolars, upper second premolar and upper first molar, upper first and second molars, and upper second and third molars were obtained (CC4-5, CC5-6, CC6-7, and CC7-8, respectively).

**Figure 2:**
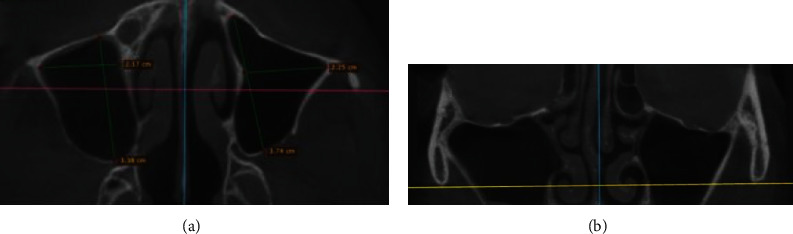
(a) Anteroposterior and mediolateral (1 and 3) measurements conducted along the root of zygoma level (AP ZG, ML ZG) on axial CBCT scan. (b) Coronal CBCT scan show the axial orientation axis (yellow horizontal line) denoting the level of axial scan along the root of zygoma.

(2) Anteroposterior (AP) and mediolateral dimension (ML) (Figure 2)

Measurements were carried out at two levels: along the nasal floor and along the root of zygoma resulting in a total of 4 different measurements (AP Nasal, AP Zygoma, ML Nasal, and ML Zygoma), respectively, on each side.

### 2.2. Sinus Volume Determination

Volumetric analysis was conducted for all the sinuses utilized to obtain linear measurements. The pipeline process of isolating the 3D volume of each sinus is described below.

**Figure 3:**
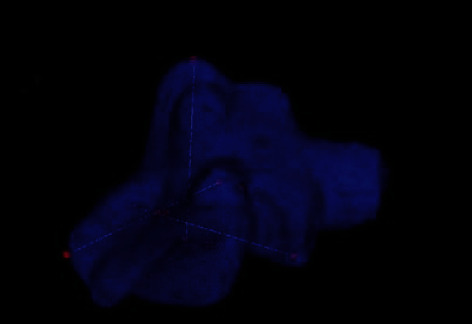
Snapshot showing measurements and calculation of the sinus volume, performed by the 3D modelling software [11].

- In each set of images, the range of image slices attached with sinus volume was isolated manually by looking at the complete range of images
- To separate the sinus region from other parts of each image, an identified threshold was assigned. The separated sinus area was coloured black, while the background was set to white
- A 3D wireframe was generated using the threshold applied images as shown in Figure 3. The covering volume was calculated with the information available from the input DICOM files. The pixel to millimeter ratio of image slices was found as 1 : 0.4726564

## 3. Results

All linear and volumetric measurements were performed by two investigator separately. To assess intraexaminer reliability, 10 maxillary sinuses were randomly selected, and all measurements were repeated. These measurements were made after a reasonable time interval. The two sets of measurements were then compared with paired *t*-tests. Out of twenty CBCT images analyzing maxillary sinuses, the male and female distribution was equal, with a mean age of 24.9 ± 3.26 years.

### 3.1. Linear Measurements of the Maxillary Sinus Height (CC)

The maximum CC dimension of the maxillary sinus was observed in between the 1^st^ and the 2^nd^ molar (CC6-7) with a mean measurement of 31.71 ± 5.44 mm, and 50% of the cases had the maximum CC dimensions at the CC 6-7. The measurements at CC 6-7 ranged from 24.6 mm to 40.9 mm on the left side with a mean of 33.09 ± 5.71 mm and 25.1 mm to 37.3 mm on the right side with a mean value of 31.24 ± 4.09 mm. In almost all cases, the maxillary sinus showed the least CC extension between the 1^st^ and the 2^nd^ premolar (CC 4-5) with a mean of 24.85 ± 4.72 mm for the left side and 23.76 ± 4.89 mm on the right side (Table 1). However, it was interesting to note that at all levels, the mean craniocaudal dimensions of the females were lesser than the mean values of the males, and the difference between the two genders in maximum mean dimension at the 1^st^ and the 2^nd^ molar (CC6-7) was statistically significant at a *P* value of 0.02 (*P* < 0.05), but the minimum mean dimensions at the 1^st^ and the 2^nd^ premolar (CC 4-5) was not statistically significant (*P* value of 0.18).

### 3.2. Linear Measurements of the Maxillary Sinus Length and Width (AP and ML)

The maximum mean value for the anteroposterior and mediolateral measurements was 32.92 ± 4.31 mm and 21.28 ± 5.09 mm, respectively, at the root of the zygoma. Out of all four measurements to assess the length and height of the sinus, it was noted that bilaterally, the maximum mean length was observed at the root of the zygoma antero-posteriorly (right side 32.02 ± 5.02 mm; left side 33.82 ± 3.48 mm; *P* = 0.36). The maximum mean height in ML direction was again at the root of the zygoma (right side 21.24 ± 5.42 mm; left side 21.33 ± 5.02 mm; *P* = 0.96). The minimum mean height was observed mediolaterally and bilaterally along the nasal floor. However, none of the differences observed on the right and left side was statistically significant at *P* < 0.05 (Table 1). In contrast to the CC measurements, all the mean of the lengths and heights were higher in sinuses belonging to females compared with the males.

The mean maximum and minimum measurements were comparable within both genders with the general findings and were on par with the craniocaudal measurements. Further, neither the maximum nor the minimum measurement differences with regard to gender showed a statistically significant difference at *P* value of 0.05 (Table 2).

### 3.3. Volumetric Analysis (Figure 4)

With regard to the measurement of the volume, the average volume of the right side was slightly more than the average value of the left side (right side—14.77 ± 4.55 cm^3^; left side—14.74 ± 4.15 cm^3^), and the males had higher values compared to the females. However, none of these differences was statistically significant (*P* = 0.89).

## 4. Discussion

There is great variation of the pneumatization of the maxillary sinus within the maxilla. This complexity is brought about as a result of differences in the extension of the maxillary sinus. Therefore, the relationship of the sinus floor with the teeth and the alveolar bone in dentate and edentulous ridge, respectively, is very crucial in the decision-making for variety of dental procedures. Such procedures may range from a simple extraction of a maxillary posterior teeth to surgical interventions including oral surgical procedures such as performing a sinus floor augmentation and planning of a dental implant in the posterior maxilla. The outcome of the dental procedure may largely depend on the assessment of volume of the maxillary sinus, and when one fails, it may give rise to a number of complications such as perforation of the sinus floor or may end up with chronic sinusitis. Literature reports seem to be controversial with regard to the changes in the size of the maxillary sinus following dental extractions where some authors witnessed an increase in the size of the maxillary sinus following tooth extraction [13]. Others oppose this fact claiming that there is no such correlation between the dental status and the volume of the maxillary sinus [14]. However, Sharah et al. in 2008 [13] identified that postextraction pneumatization occurred within the socket healing period of 4 to 6 months, and this process may reduce to a minimum or stop completely following the development of mature bone in the extraction socket.

According to the 3D volumetric segmentation, demonstrated by Hamdy et al., in 2012 [15], the linear measurements performed in this study represent the width, length, and the height of the sinus (from the measurements done on mediolateral anteroposterior and craniocaudal direction, respectively). A retrospective study by Hamdy and Abdel-Wahed [12], analyzing 30 CBCT scans reported that the maximum craniocaudal, mediolateral, and anteroposterior dimensions were observed around 2^nd^ molar (93%) and at the level of the root of zygomatic complex in 93% and 90% sinuses, respectively. These findings were comparable with results of the present study. In this study, it was found that the largest average craniocaudal, mediolateral, and anteroposterior extensions of the maxillary sinus using CBCT were 31.71 ± 5.44 mm, 21.28 ± 5.09 mm, and 32.92 ± 4.31 mm, respectively. Our results demonstrated that the height of this pyramid (mediolateral) is the smallest dimension, while the anteroposterior and craniocaudal dimensions of its base are nearly equal. These findings are comparable with the average dimensions reported by the sinus studies by Tiwana et al. [16] and Hamdy and Abdel-Wahed et al. [12]. Further, according to Tiwana et al., the average dimensions of the sinus are 33 mm high, 23 mm wide, and 34 mm in anterior-posterior length [16].

Further, the results obtained in the present study demonstrated that only 50% of the cases had the maximum craniocaudal extension of the maxillary sinus located around the 1st and 2nd molar (10 cases out of 20 = 50%). This result is similar to the studies by Koppe et al. [17] who observed prominences on maxillary sinus floor due to the apices of the first and second maxillary molars in half of its samples. Further, in par with this finding, Ariji et al. [18] reported to observe 60% of the roots of the maxillary first molar close to the sinus floor. However, this finding contrasts to the ones reported by Nimigean et al. [19], as he reported in majority the lowest point of the maxillary sinus is in relation to the second molar (93.9%). The variations of the sinus floor's depth will depend on sinus dimensions, their size, and pneumatization [19].

A study conducted by Saccucci et al. [20] to determine the relationship between gender and maxillary sinus volumes included 52 patients (26 males and 26 females) with a mean age of 24.3 years revealed that there is no statistically significant difference in the patients' maxillary sinus volumes across genders. The results of our study also confirm this fact as there was no statistically significant difference in the linear and volumetric measurements except in linear measurement (craniocaudal) between the 1st and 2nd molar teeth. However, a study by Georgiev et al. [21] has revealed a statistically significant difference in favor of male patients in their analysis of volumetric measurements across genders.

The present study, based on a special software application, facilitated a quick and easy estimate of the volume of the maxillary sinus, which has an irregular shape and thus defies accurate mathematical calculation. The volumetric assessment of the maxillary sinus and its pneumatization (primary or secondary) allows clinicians to accurately determine the exact site of surgical intervention and its anatomical variation. Maxillary sinus floor augmentation procedures with lateral access require precise location of the bone window as well as a sufficiently wide sinus, so that elevation or removal could be adequate, and determination of the volume of bone graft material is required for sinus grafting procedures.

## 5. Conclusions

Within the limitations of the present study, the findings highlighted the anatomic variability of the maxillary sinus in relation to several parameters. In comparison to the figures reported in literature, certain aspects of the morphometrics of maxillary sinus in a Sri Lankan population are different from the standard measurements reported for other population groups. Utilization of a cost-effective software for the volumetric assessment is also encouraged especially in the Sri Lankan context. Further studies with larger numbers of CBCT images covering all age groups and ethnic groups will be needed for more information in the Sri Lankan population.

## Figures and Tables

**Figure 4:**
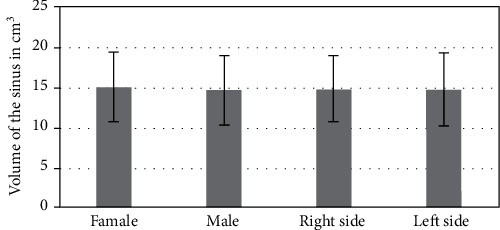
Mean volume of sinuses against the side and gender.

**Table 1:** Linear measurements of the maxillary sinus, width, length, and height-denoting extension in CC, AP, and ML directions at different levels.

	Left side								Right side							
	Maxillary sinus width				Maxillary sinus height and length				Maxillary sinus width				Maxillary sinus height and length			
	CC 4-5	CC 5-6	CC 6-7	CC 7-8	ML NS	ML ZG	AP NS	AP ZG	CC 4-5	CC 5-6	CC 6-7	CC 7-8	ML NS	ML ZG	AP NS	AP ZG
01	31.3	36.1	34.6	36.8	22.6	24.4	36.1	37.3	32.6	37.5	37.3	36.2	21.3	23.2	31.3	34.9
02	21.8	36	35.5	34.3	14.3	20	28.3	30	26.4	31.7	33.2	37.5	13.1	19.6	20.2	24.2
03	25.3	37.8	40.9	35.9	18.4	20	32.6	33.9	22.2	34.5	33.5	23.2	15.7	19.4	24.9	26.6
04	26.2	33.4	38.7	34.2	23.6	28.5	35.4	38.5	21.8	33.1	34	34	20.5	26.3	35.2	37.8
05	23.7	26.8	24.6	30.8	14.5	13	11.9	29.2	18.4	28	31.1	27.2	11.8	14.1	12.1	30.6
06	29.6	28.2	28.4	30.8	16.6	23.2	35.4	36.4	27.9	26.1	29.6	33.1	16.1	23.9	32.1	38.7
07	30.4	35.7	40.3	34.7	20.5	22.2	36.2	36.2	28.8	34.7	35.2	30.9	17.4	22.4	37	36.6
08	19.7	26.5	28.9	28.7	18.6	17.4	23.4	30.2	18.6	22.1	26.5	26.7	17.5	15.7	21.5	26.8
09	16.8	26.1	28.2	25.9	14	16.3	28.5	30.7	19.1	27	25.1	26.6	10.5	16	22.2	31.7
10	23.7	22.7	30.8	28.4	10.5	28.3	25.1	35.8	21.8	24.2	26.9	26.3	20.4	31.8	24.7	32.3
Mean	24.85	30.93	33.09	32.05	17.36	21.33	29.29	33.82	23.76	29.89	31.24	30.17	16.43	21.24	26.12	32.02
SD	4.72	5.41	5.71	3.64	4.15	5.02	7.72	3.48	4.89	5.11	4.09	4.84	3.75	5.42	7.71	5.02
95% CI	3.01	3.45	3.64	2.32	2.97	3.59	5.52	2.49	3.49	3.66	2.93	3.47	2.68	3.88	5.52	3.59

**Table 2:** The maximum and minimum linear measurements of the maxillary sinuses against the gender.

	Maxillary sinus width		Maxillary sinus length		Maxillary sinus height	
	Maximum mean ± SD (mm)	Minimum mean ± SD (mm)	Maximum mean ± SD (mm)	Minimum mean ± SD (mm)	Maximum mean ± SD (mm)	Minimum mean ± SD (mm)
Male	34.59 ± 4.49	25.71 ± 5.00	32.02 ± 5.02	26.12 ± 7.71	21.24 ± 5.42	16.43 ± 3.75
Female	29.74 ± 4.24	22.90 ± 4.17	33.82 ± 3.48	29.29 ± 7.72	21.33 ± 5.02	17.36 ± 4.15
*P* value	0.02	0.18	0.99	0.37	0.97	0.6

SD: standard deviation; mm: millimeters.

## Data Availability

Access to research data is restricted as these contain patient records.
